# LPS-Induced Neuroinflammation Increases Serotonin-Evoked Activity of Trigeminal Afferents and Aggravates Mechanical Allodynia and Photophobic Behavior in Rat Migraine Model

**DOI:** 10.3390/ijms262411983

**Published:** 2025-12-12

**Authors:** Svetlana Svitko, Elisaveta Ermakova, Karina Gilizhdinova, Ksenia Bogatova, Nazgul Gaifutdinova, Dinara Nurmieva, Egor Nevsky, Anton Ananev, Olga Yakovleva, Albert Sufianov, Galina Z. Sufianova, Artyom Baev, Kseniia Shaidullova, Albert Rizvanov, Aliya Yakubova, Guzel Sitdikova

**Affiliations:** 1 Department of Human and Animal Physiology, Institute of Fundamental Medicine and Biology, Kazan Federal University, 420008 Kazan, Russia; 2 Petrovsky National Research Center of Surgery, 119435 Moscow, Russia; 3 The Research and Educational Institute of Neurosurgery, Peoples’ Friendship University of Russia (RUDN), 117198 Moscow, Russia; 4 Department of Pharmacology, Tyumen State Medical University, 625023 Tyumen, Russia; 5 Laboratory of Experimental Biophysics, Centre for Advanced Technologies, Tashkent 100174, Uzbekistan; 6 Division of Medical and Biological Sciences, Tatarstan Academy of Sciences, 420111 Kazan, Russia; 7 Department of Gene and Cell Technology, Institute of Fundamental Medicine and Biology, Kazan Federal University, 420021 Kazan, Russia; 8 Department of Pathophysiology, Kyrgyz State Medical Academy, Bishkek 720020, Kyrgyzstan

**Keywords:** neuroinflammation, migraine, sensitization, LPS, trigeminal system, mechanical allodynia, photophobia, mast cell, CGRP

## Abstract

Migraine is characterized by severe pain and somatic symptoms like allodynia and photophobia, driven by neuroinflammation that sensitizes the trigeminal vascular system (TVS). This study investigated how neuroinflammation induced by systemic lipopolysaccharide (LPS) affects migraine-related nociceptive signaling. Using a chronic migraine model in rats with nitroglycerin (NTG), we compared prenatal and acute postnatal LPS administration. Rats with prenatal LPS exhibited lower mechanical thresholds and enhanced allodynia and photophobia after NTG. Acute LPS also increased allodynia, but not photophobia. Both LPS groups showed increased mast cell degranulation in the dura mater. Plasma CGRP after NTG administration was elevated in the acute LPS group. Electrophysiology revealed enhanced trigeminal afferent responses to serotonin in both acutely and prenatally LPS-treated rats. Calcium imaging demonstrated increased neuronal responses to serotonin and capsaicin, suggesting an upregulation of serotonin and TRPV1 receptors. Our findings show that LPS-induced neuroinflammation, whether prenatal or acute, promotes sensitization of peripheral and central nociceptive pathways, involving serotoninergic mechanisms.

## 1. Introduction

Migraine is a heterogeneous disease characterized by recurrent intense headaches, which may be accompanied by neurological symptoms and changes in sensory perception (known as an aura), as well as various somatic symptoms [1,2,3]. Migraine affects 15–18% of the world’s population and is considered the most disabling neurological disorder, due to its significant negative impact on patients’ quality of life and ability to work [4,5]. The high prevalence of migraine among young people of working age, as well as the intensity of the pain syndrome, place a significant burden on the economy and healthcare system [5,6].

According to current knowledge, the mechanism of headache onset in migraine involves the activation of peripheral structures of the trigeminal vascular system (TVS), which includes the dura mater and the meningeal vessels innervated by nociceptive afferents of the trigeminal ganglion (TG) [1,3,7]. Various mediators, such as nitric oxide, calcitonin gene-related peptide (CGRP), extracellular adenosine triphosphate (ATP), serotonin, and others, can lead to the generation of pain signals [1,3,8,9]. The sources of pro-nociceptive mediators released in the TVS may be blood vessels, nerve fibers, and mast cells located in the dura mater, which are capable of forming the so-called neuro-immune synapse [10,11,12,13]. Moreover, the degranulation of mast cells in the dura mater (along with activation of microglia) may mediate the neuroinflammatory component of migraine pathogenesis, which is particularly pronounced in its chronic form [14,15]. Recent genetic findings suggest that alterations in the genes responsible for inflammation and metabolism are implicated in the pathogenesis of chronic migraine [16]. Neuroinflammation is an inflammatory process in the brain and spinal cord mediated by the production of cytokines, chemokines, reactive oxygen species, and secondary messengers [14,15,17,18,19]. Neuroinflammation is characterized by increased blood–brain barrier permeability, extravasation of immune cells into the brain parenchyma, and the activation of microglia. The role of neuroinflammation in the development of neurological diseases has been established: Alzheimer’s disease [20], Parkinson’s disease [21], lateral sclerosis [22], etc.

The role of neuroinflammation in migraine mechanisms is also under active investigation [11,12,13,14,15,23]. It is believed that mast cell degranulation in the dura mater is one of the factors contributing to the peripheral sensitization of trigeminal meningeal afferents [1,11,12,13,14,15]. The phenomenon of peripheral sensitization consists of the increased sensitivity of meningeal afferents resulting from the action of pro-nociceptive compounds, as well as cytokines and chemokines released from mast cells and macrophages [14,15]. Neuroinflammatory reactions play a key role in peripheral sensitization, creating a feedback loop of nociceptive signal amplification as a result of interactions between immune cells and nerve endings [10,11,12,14,15].

The most common somatic symptoms specific to migraine include hypersensitivity to light (photophobia), tactile allodynia, as well as nausea, vomiting, fatigue, and increased sleepiness [1,10,24,25]. It is believed that various somatic symptoms in migraine may be caused by both the activation of peripheral parts of the trigeminal vascular system and the involvement of central structures, such as the trigeminal nucleus caudalis (TNC), which has ascending and descending connections with the thalamic and cortical regions [1,10]. It is known that sensitization of the meningeal afferents of the trigeminal nerve may be followed by sensitization in the central structures, which mediates the amplification of nociceptive signals transmitted from the periphery and the manifestation or intensification of many somatic symptoms of migraine [1,14]. Currently, despite existing advances in understanding the fundamental mechanisms of migraine pathogenesis, the details of the molecular and cellular mechanisms of nociceptive signaling in the trigeminal vascular system remain poorly understood, complicating the development of new and more effective therapeutic agents for this disease.

Lipopolysaccharide (LPS, an endotoxin found in the cell wall of Gram-negative bacteria) administration is widely used to create a model of inflammatory, post-traumatic, postoperative, or neuropathic pain for further research into nociception-related molecular mechanisms and neuronal pathways [26,27,28,29]. However, to date, there are no experimental studies devoted to assessing the role of neuroinflammation caused by LPS administration on nociceptive processes in migraine, especially using an NTG-induced rodent migraine model, although a number of studies indicate that LPS administration may apparently contribute to the intensification of nociceptive responses [29], including the development of hyperalgesia [26,27,28,29,30,31,32,33,34], which can potentially worsen the course of migraine.

In this study, we used LPS administration during pregnancy for the first time to assess the effects of prenatally induced inflammation on offspring behavior in the migraine model, induced by nitroglycerine (NTG) administration, and on neuroinflammatory responses of mast cells in the dura mater. We further evaluated the role of the systemic inflammation induced by acute LPS administration on the behavioral correlates of migraine, as well as on the degranulation of meningeal mast cells. In addition, using an electrophysiological approach and Ca^2+^ imaging, we assessed the electrical activity of trigeminal afferents and Ca^2+^ signaling in isolated neurons of TG in response to LPS-induced neuroinflammation.

## 2. Results

### 2.1. Mechanical Sensitivity of Rats in Models of Prenatal and Acute LPS-Induced Inflammation Following Single NTG Administration

**Figure 1:**
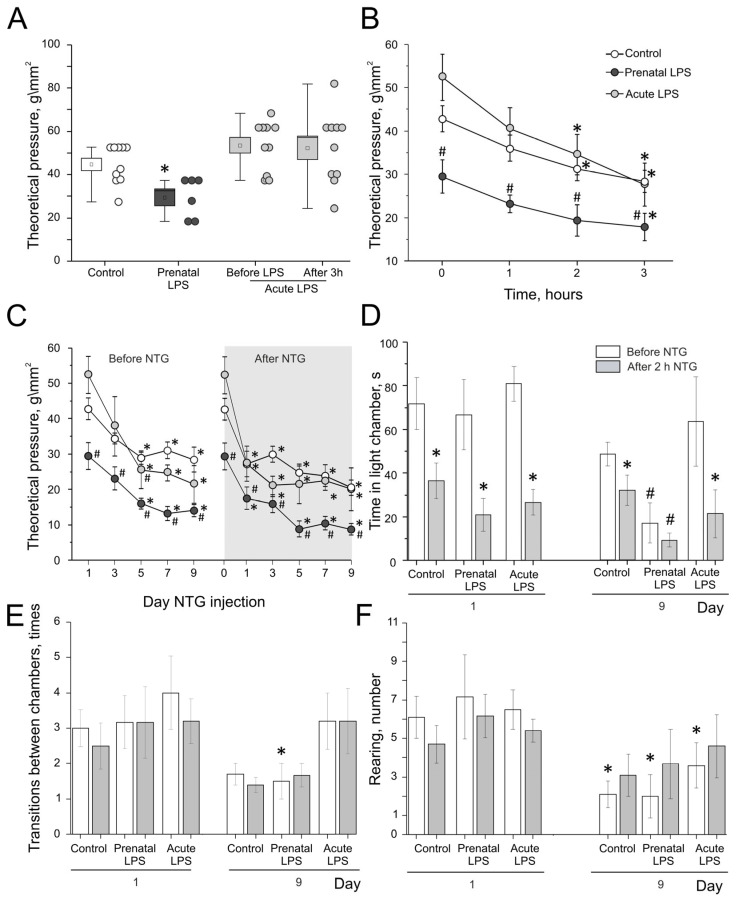
Behavioral changes in episodic and chronic migraine model. (**A**) Mechanical thresholds in the control group (white) and prenatal LPS (dark gray), before and after LPS administration (acute LPS, light gray), small dots next to boxplots represent data for each experiment; (**B**) threshold of mechanical sensitivity before and after 1, 2, and 3 h of NTG administration in episodic migraine model of the control (white), prenatal LPS (dark gray), and acute LPS (light gray) groups; (**C**) pre- (before NTG) and post-injectional (after NTG) mechanical thresholds during chronic administration of NTG of the control (white), prenatal LPS (dark gray), and acute LPS (light gray) groups; (**D**) time spent in light chamber before (white columns) and 2 h after (gray columns) NTG administration on first and ninth days of chronic injections of the control, prenatal LPS, and acute LPS groups; (**E**) number of transitions between chambers before (white columns) and 2 h after (gray columns) NTG administration on first and ninth days of chronic injections of the control, prenatal LPS, and acute LPS groups; (**F**) rearing count before (white columns) and 2 h after (gray columns) NTG administration on first and ninth days of chronic injections of the control, prenatal LPS, and acute LPS groups. * *p* < 0.05 compared to initial value; # *p* < 0.05 compared to the control group.

The initial mechanical threshold of the hind paw of control rats (n = 10) was 43.9 ± 3.3 g/mm^2^, while the mechanical threshold of rats from the prenatal LPS group (n = 6) was significantly lower—29.5 ± 3.8 g/mm^2^ (*p* = 0.008; Figure 1A). In rats with acute administration of LPS (n = 10), the initial threshold was 52.5 ± 4.2 g/mm^2^, and 3 h after LPS injection, it was 52.4 ± 5.3 g/mm^2^ (*p* = 0.9) and did not differ from the values of the control group (*p* = 0.2; Figure 1A).

A single administration of NTG widely used to model the behavioral correlates of episodic migraine, induced a decrease in the mechanical thresholds measured 1, 2, and 3 h after injection, which is consistent with the previously obtained data [35,36]. In the control group one hour after NTG administration, the mechanical threshold of the hind paws did not significantly differ (from 43.9 ± 3.3 g/mm^2^ to 37.2 ± 3.0 g/mm^2^; *p* = 0.2; Figure 1B). Two hours after NTG administration, the mechanical thresholds significantly decreased to 32.6 ± 2.8 g/mm^2^ (*p* = 0.03), and three hours after injection, they decreased to 27.4 ± 3.6 g/mm^2^ (*p* = 0.01).

In the sham group, the mechanical thresholds were measured after the injection of saline and did not change during 3 h. The initial value was 45 ± 4.4 g/mm^2^, one hour after injection, it was 64.7 ± 8.75 g/mm^2^, and it remained stable 2 and 3 h later (64.7 ± 8.75 g/mm^2^ and 64.7 ± 8.75 g/mm^2^, respectively).

In the prenatal LPS group, the mechanical thresholds significantly decreased after 3 h of NTG injections from 29.5 ± 3.8 g/mm^2^ to 17.8 ± 3.1 g/mm^2^ (*p* = 0.04; Figure 1B).

In the acute LPS group, the dynamics of the mechanical thresholds after NTG injection were similar to the control group, and a significant decrease was observed two hours after injection, from 52.4 ± 5.3 g/mm^2^ to 34.5 ± 4.7 g/mm^2^ (*p* = 0.02), and after 3 h, it was 27.6 ± 4.9 g/mm^2^ (*p* = 0.01; Figure 1B).

### 2.2. Pre-Injectional Mechanical Thresholds During Chronic NTG Administration

In the sham group (n = 4), the values of the mechanical threshold did not differ from the initial ones; on day 1 of saline injections, it was 45 ± 4.4 g/mm^2^, on day 3, it was 50.3 ± 6.6 g/mm^2^, on day 5, it was 42.6 ± 3.4 g/mm^2^, on day 7, it was 45.7 ± 4 g/mm^2^, and on day 9, it was 42.5 ± 8.6 g/mm^2^.

In the control group (n = 10), the initial value of the mechanical threshold was 52.4 ± 5.3 g/mm^2^. Before the second injection of NTG (on the day 3 of the experimental procedures), the basal threshold significantly decreased to 35.1 ± 4.5 g/mm^2^ (*p* = 0.03); on day 9, it was 31 ± 3.5 g/mm^2^ (*p* = 0.02; Figure 1C).

In the prenatal LPS group (n = 6), the pre-injection mechanical threshold before the second NTG administration decreased from 29.5 ± 3.8 g/mm^2^ (the initial basal threshold) to 23.1 ± 3.2 g/mm^2^ (*p* = 0.25); before the third injection, it was 16 ± 1.5 (*p* = 0.015), before the fourth injection, it was 13.2 ± 2 g/mm^2^ (*p* = 0.01), and before the fifth injection, it was 14 ± 1.7 g/mm^2^ (*p* = 0.001; Figure 1C).

In the acute LPS group (n = 5), the pre-injection mechanical thresholds decreased from 52.5 ± 4.2 g/mm^2^ to 37.8 ± 8.4 g/mm^2^ (*p* = 0.2) before the second NTG injection. Before the third NTG injection, the pre-injectional threshold was 21 ± 2.9 g/mm^2^ (*p* = 0.01), which was significantly lower than the control group (*p* = 0.008), and it remained at the same level before the fifth NTG injection—21.4 ± 5.3 g/mm^2^ (*p* = 0.01; Figure 1C).

### 2.3. Post-Injectional Mechanical Thresholds After Chronic Administration of NTG

Next, we analyzed the post-injection mechanical thresholds 3 h after NTG administration on days 3, 5, 7, and 9, and the obtained parameters were compared with the basal pre-injection thresholds before the first NTG injection.

In the sham group (n = 4), five injections of saline did not change mechanical thresholds significantly. The initial value was 45 ± 4.4 g/mm^2^, and the mechanical thresholds after the first, second, third, fourth, and fifth injections were 64.7 ± 8.75 40.3 ± 5 g/mm^2^; 42.6 ± 3.4 g/mm^2^, 42.6 ± 3.4 g/mm^2^, and 42.5 ± 8.6 g/mm^2^, respectively (all *p* > 0.05 compared to initial value).

In the control group (n = 10), after the first NTG administration, the mechanical thresholds decreased from 43.9 ± 3.3 g/mm^2^ to 27.4 ± 3.6 g/mm^2^ (*p* = 0.01); after the second injection, they were 31.9 ± 2.3 g/mm^2^ (*p* = 0.01); after the third injection, they decreased to 25.8 ± 2.4 g/mm^2^ (*p* = 0.002); after the fourth injection, they were 25.9 ± 2.9 g/mm^2^ (*p* = 0.003); and after the fifth injection, they decreased to 23.4 ± 1.4 g/mm^2^ (*p* = 0.003) (Figure 1C).

In the prenatal LPS group (n = 6), after the first day of NTG administration, the mechanical thresholds significantly decreased from 29.5 ± 3.8 g/mm^2^ to 17.8 ± 3.1 g/mm^2^ (*p* = 0.04); after the second injection, they decreased to 16.1 ± 2.5 g/mm^2^ (*p* = 0.02); after the third, they decreased to 8.9 ± 2.3 g/mm^2^ (*p* = 0.004); after the fourth, they were 10.6 ± 1.8 g/mm^2^ (*p* = 0.004); and after the fifth, they decreased to 8.8 ± 1.6 g/mm^2^ (*p* = 0.004) (Figure 1C).

In the acute LPS group (n = 10), after the first NTG injection, the mechanical threshold decreased from 52.4 ± 5.3 g/mm^2^ to 27.6 ± 4.9 g/mm^2^ (*p* = 0.02); after the second injection, it decreased to 24.6 ± 2.2 g/mm^2^ (n = 5, *p* = 0.01), and this value was significantly lower than the control group (*p* = 0.01). After the third, fourth, and fifth NTG injections, the mechanical thresholds were 22.3 ± 2.3 g/mm^2^ (*p* = 0.01), 26.9 ± 4.7 g/mm^2^ (*p* = 0.025), and 20.2 ± 6.1 g/mm^2^ (*p* = 0.01), respectively (Figure 1C).

### 2.4. Effects of Prenatal and Acute Administration of LPS on Photophobic Behavior and Locomotor Activity in the Light–Dark Transition Test in a Model of Episodic and Chronic Migraine

Light avoiding behavior (photophobia) is a specific behavioral sign of migraine. The amount of time spent in a light chamber was measured on days 1 and 9 of NTG administration (or saline in the sham group) before and 2 h after injection. In the sham group (n = 4), on day 1 of saline administration, animals spent 82.5 ± 9 s and 79 ± 43.1 s in the light chamber, respectively. On day 9 of saline administration, before injection, animals spent 57.5 ± 41.1 s in the light chamber and 88 ± 34.15 s 2 h after the injection. Thus, with the administration of saline, there was no decrease in the amount of time spent in the light chamber after the injection, indicating the absence of photophobic behavior. In the control group (n = 10), at day 1 of NTG administration, before injection, animals spent 71.8 ± 12 s in the light chamber and significantly less time, 36.5 ± 8.1 s 2 h, after the injection (*p* = 0.02; Figure 1D). On day 9 of NTG administration, before injection, animals spent 51.1 ± 5.1 s in the light chamber and significantly less time, 29.8 ± 7.2 s, 2 h after the injection (*p* = 0.01), which did not differ from values of first day before (*p* = 0.09) and after the injection (*p* = 0.8).

In the prenatal LPS group (n = 6), on day 1 of NTG administration, before injection, animals spent 66.7 ± 16.1 s in the light chamber, and significantly less time, 20.8 ± 7.4 s, 2 h after NTG injection (*p* = 0.004; Figure 1D). On day 9 of NTG administration, before injection, animals spent only 17 ± 9.2 s, which is significantly less than before injection at the day 1 (*p* = 0.045) and less than the animals in the control group (*p* = 0.02). Two hours after the injection, they spent less time, 9.2 ± 3.1 s, in the light chamber. This value is significantly lower than the control group (9.2 ± 3.1 s vs. 29.8 ± 7.2 s, *p* = 0.02).

In the acute LPS group (n = 10), on day 1 of NTG administration, before injection, animals spent 80.8 ± 8 s in the light chamber and significantly less time, 26.7 ± 5.9 s (*p* = 0.003), 2 h after NTG injection (Figure 1D). This post-injection indicator did not differ from the similar indicator in the control group (*p* = 0.4). On day 9 of NTG administration, before injection, animals (n = 5) spent 63.7± 20.5 s and significantly less time, 21.3 ± 11.1 s (*p* = 0.04), 2 h after the injection, which did not differ from the values of first day before (*p* = 0.7) and after the injection (*p* = 0.5).

Evaluation of the locomotor activity in each group showed that the NTG injections did not affect the rearing count and number of transitions between chambers. In the sham group (n = 4), on the first day of NaCl administration, we observed 5.5 ± 0.3 transitions between chambers, and 2 h after saline injection, the number of transitions was 4.25 ± 0.5. On day 9 of saline administration, before the injection, the number of transitions was 4.5 ± 0.9, and after the injection, it was 3.75 ± 0.5. In the control group (n = 10), on day 1 of NTG administration, before the injection, we observed 3 ± 0.5 transitions between chambers, and after the NTG injection, it was 2.5 ± 0.65 (*p* = 0.3 compared to the rearing count before injection; Figure 1E). On day 9 of NTG administration, before injection, the number of transitions was 1.7 ± 0.3, and after NTG administration, it was 1.4 ± 0.2. In the prenatal LPS group (n = 6), on day 1 of NTG administration, before the injection, the number of transitions was 3.2 ± 0.7, and after the injection, it was 3.2 ± 1.65 (*p* = 1 compared to the number of transitions before injection). On day 9 of NTG administration, before injection, the number of transitions was 1.5 ± 0.5 (*p* = 0.03 compared to the pre-injection transitions number on day 1; Figure 1E), and after NTG injection, it was 1.7 ± 0.3. In the acute LPS group (n = 10), on day 1 of NTG administration, before the injection, we observed 4 ± 1 transitions, and 2 h after injection, this parameter was 3.2 ± 0.6 (*p* = 0.8 compared to number of transitions before injection). On day 9 of NTG administration, before injection, the number of transitions was 3.2 ± 0.8, and after the injection, it was 3.2 ± 0.9.

In the sham group (n = 4), on the first day of NaCl administration, we observed an 11 ± 1.2 rearing count, and it was 8.5 ± 0.6 after the injection. On the ninth day of saline administration, before the injection, the rearing count was 7.5 ± 2.1, and after the injection, it was 7 ± 1.4. In the control group (n = 10), on day 1 of NTG administration, before injection, the rearing count was 6.1 ± 1.1, and after the NTG injection, it was 4.7 ± 1 (*p* = 0.4 compared to the rearing count before injection; Figure 1F). On day 9 of NTG administration, before injection, the rearing count was 2.1 ± 0.7 (*p* = 0.02 compared to the pre-injection rearing count on the first day; Figure 1F), and 2 h after NTG injection, it was 3.1 ± 1. In the prenatal LPS group (n = 6), on day 1 of NTG administration, before injection, the rearing count was 7.2 ± 2.2, and 2 h after the injection, it was 6.2 ± 1.1 (*p* = 0.7 compared to the rearing count before injection). On day 9 of NTG administration, the rearing count was 2 ± 1.1 (*p* = 0.01 compared to the pre-injection rearing count on the first day), and after the injection, it was 3.7 ± 1.8. In the acute LPS group (n = 10), on day 1 of NTG administration, before injection, the rearing count was 6.5 ± 1, and 2 h after the injection, the rearing count was 5.4 ± 0.6 (*p* = 0.85 compared to the rearing count before injection). On day 9 of NTG administration, the rearing count was 3.6 ± 1.2 (*p* = 0.04 compared to the pre-injection rearing count on the first day; Figure 1F), and 2 h after the injection, it was 4.6 ± 1.6.

### 2.5. Mast Cell Degranulation in the Dura Mater

**Figure 2:**
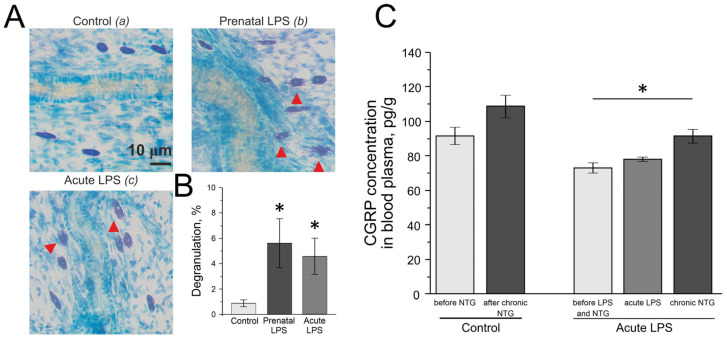
Mast cell degranulation in rat meninges and CGRP level in plasma. (**A**) Toluidine Blue staining of the meninges in the control (**a**), prenatal LPS (**b**), and acute LPS (**c**) groups; red arrow points to degranulated cells. (**B**) Percentage of degranulated mast cells in the control, acute LPS, and prenatal LPS groups. (**C**) CGRP level in blood plasma of the control group before (light gray) and after chronic administration of NTG (dark gray) and of the acute LPS group before (light gray) and after administration of LPS (medium gray) and LPS+NTG (dark gray). Mean ± SEM. * *p* < 0.05.

In the control group of rats (n = 10), the degranulation of meningeal mast cells was 0.88 ± 0.27%. In rats from the acute LPS group, the degranulation of meningeal mast cells was significantly higher compared to the control group—4.6 ± 1.4% (n = 5, *p* = 0.006; Figure 2A,B). In the prenatal LPS group, the meningeal mast cell degranulation was 5.6 ± 1.9% (n = 6, *p* = 0.005; Figure 2A,B).

### 2.6. Evaluation of CGRP Level in Blood Plasma of Rats with Model of Chronic Migraine and Acute or Postnatal LPS Administration

In the control group (n = 6), prior to NTG administration, the CGRP content in blood plasma was 91.5 ± 4.9 pg/mL, and after chronic NTG administration, the level of CGRP was 108.7 ± 6.5 pg/mL (*p* = 0.07), which was 119.5 ± 6.7% of the initial level (*p* = 0.06; Figure 2C).

In the acute LPS group (n = 5), the initial CGRP level in blood plasma was 73.1 ± 2.9 pg/mL. After LPS administration, this level did not change—77.9 ± 1.2 pg/mL (*p* = 0.1; Figure 2C). As a result of the five-time administration of NTG, the CGRP level increased to 91.3 ± 3.8 pg/mL (n = 5) and differed significantly from the initial CGRP level (*p* = 0.01). Compared to the initial level, the CGRP level increased to 125.6 ± 6.5% (*p* = 0.007).

### 2.7. Electrophysiological Recording of Action Potentials of Trigeminal Nerve Afferents in Acute LPS Group

**Figure 3:**
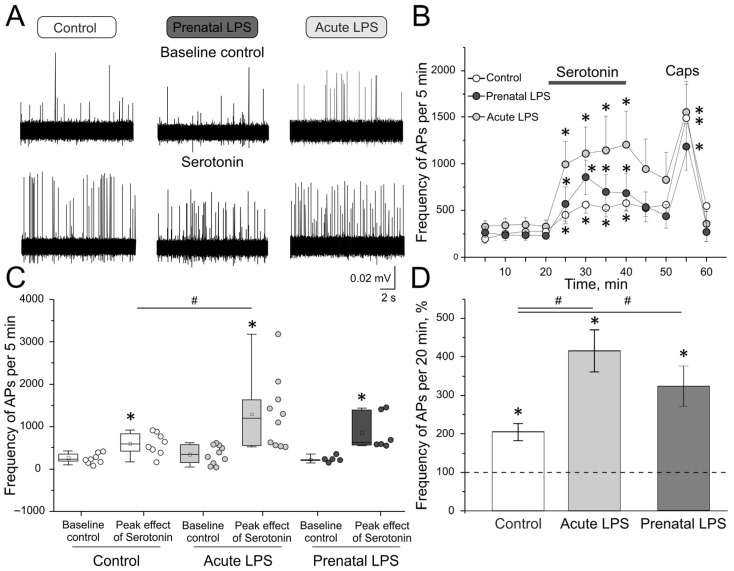
Electrical activity of trigeminal afferents in rat hemiskull preparations. (**A**) Sample traces of APs recorded in the trigeminal nerve before (baseline control) and after the application of serotonin (20 μM) in the control group, acute LPS group, and prenatal LPS group. (**B**) The time course of AP frequency after the application of serotonin and capsaicin in the control group (white circles), acute LPS group (black circles), and prenatal LPS group (gray circles). (**C**) The maximum values of APs per 5 min (peak effect) during serotonin application in the control group, in the acute LPS group, and in the prenatal LPS group; small dots next to boxplot represent data for each experiment. (**D**) Normalized effect of 20 min of serotonin application (20 μM) on the control (light grey), acute LPS (medium gray), and prenatal LPS (dark gray) groups. Dotted line represents baseline activity (100%) for 20 min before application of serotonin. * *p* < 0.05 compared to initial value; # *p* < 0.05 compared to the control group.

In the control group (n = 8), the initial number of APs was 246.7 ± 40.4 per 5 min. The application of serotonin (20 μM) led to a significant increase in the number of APs to 455.4 ± 97.3 per 5 min in the first 5 min of application (*p* = 0.03; Figure 3A,B). After 10 min of application, the number of APs was 567.8 ± 95.5 per 5 min (*p* = 0.01), after 15 min of serotonin application, the number of APs was 530.7 ± 99.7 per 5 min (*p* = 0.02), and after 20 min, it was 581.1 ± 89.7 per 5 min (*p* = 0.01). The application of capsaicin (1 μM), performed after a 10 min washout of the serotonin, led to an increase in APs frequency from 564.8 ± 91.4 per 5 min to 1495 ± 350.7 per 5 min (*p* = 0.001).

In rats from the acute LPS group (n = 10), the initial number of APs was 339.5 ± 68.9 per 5 min. When serotonin (20 μM) was applied, a significant increase in the number of APs was observed in the first 5 min of application, up to 997.7 ± 239.35 per 5 min (*p* = 0.005), similar to the control group (Figure 3A,B). After 10 min of application, the number of APs was 1113.5 ± 279.1 per 5 min (*p* = 0.005), after 15 min of application, the number of APs was 1147.2 ± 359.8 per 5 min (*p* = 0.005), and after 20 min of application, the number of APs was 1208.7 ± 352.4 per 5 min (*p* = 0.005). The application of capsaicin (1 μM), performed after a 10 min washout of the serotonin, led to an increase in number of APs from 831 ± 292.8 per 5 min to 1559.3 ± 321.6 per 5 min (*p* = 0.045).

In rats from the prenatal LPS group (n = 6) the initial number of APs was 211.2 ± 30.5 per 5 min. When serotonin (20 μM) was applied, a significant increase in the number of APs was observed in the first 5 min of application, up to 561.3 ± 184.8 per 5 min (*p* = 0.03) similar to the control group (Figure 3A,B). After 10 min of application, the number of APs was 848.2 ± 183.2 per 5 min (*p* = 0.03), after 15 min of application, the number of APs was 689.2 ± 184.7 per 5 min (*p* = 0.03), and after 20 min of applications, the number of APs was 678.5 ± 205.6 per 5 min (*p* = 0.03). The application of capsaicin (1 μM), performed after a 10 min washout of the serotonin, led to an increase in number of APs from 432.5 ± 125.3 per 5 min to 1168.3 ± 246.9 per 5 min (*p* = 0.03).

The maximum effect of serotonin in the acute LPS group was 1501.4 ± 432.7 APs in 5 min, which differed significantly from the maximum effect of serotonin in the control group—581.1 ± 89.7 APs in 5 min (*p* = 0.04; Figure 3C). In rats from the prenatal LPS group, the maximum effect was 837.7 ± 175.2 APs in 5 min and was not different from the control group (*p* = 0.3).

After that, we calculated the number of APs during 20 min of serotonin (20 μM) application and normalized it to 20 min of the recording of the initial activity (Figure 3D). In the control group, the number of APs before application of the substance was 986.7 ± 161.7 in 20 min; as a result of 20 min of serotonin application, the number of APs increased to 2022 ± 334.3 in 20 min (*p* = 0.01), which is an increase of 205.3 ± 22.2% relative to the number of APs before application (*p* = 0.01; Figure 3D).

In the acute LPS group, the number of APs before serotonin application was 1272.25 ± 325.3 per 20 min. As a result of 20 min of serotonin application, the number of APs increased to 4758.9 ± 1458.7 per 20 min (*p* = 0.006). After normalization, it was found that 20 min of serotonin application led to a significant increase in the number of APs by 415.9 ± 54.6% (*p* = 0.01), which was significantly different from the normalized number of APs in 20 min with serotonin application in the control group (*p* = 0.01; Figure 3D).

In the prenatal LPS group, the number of APs before serotonin application was 883.2 ± 246.5 per 20 min. As a result of 20 min of serotonin application, the number of APs increased to 2777.2 ± 336.5 per 20 min (*p* = 0.03). After normalization, it was found that 20 min of serotonin application led to a significant increase in the number of APs by 322.7 ± 53.1% (*p* = 0.03), which was significantly different from the normalized number of APs in 20 min with serotonin application in the control group (*p* = 0.02; Figure 3D).

### 2.8. Ca^2+^ Imaging in Trigeminal Ganglion Neurons

**Figure 4:**
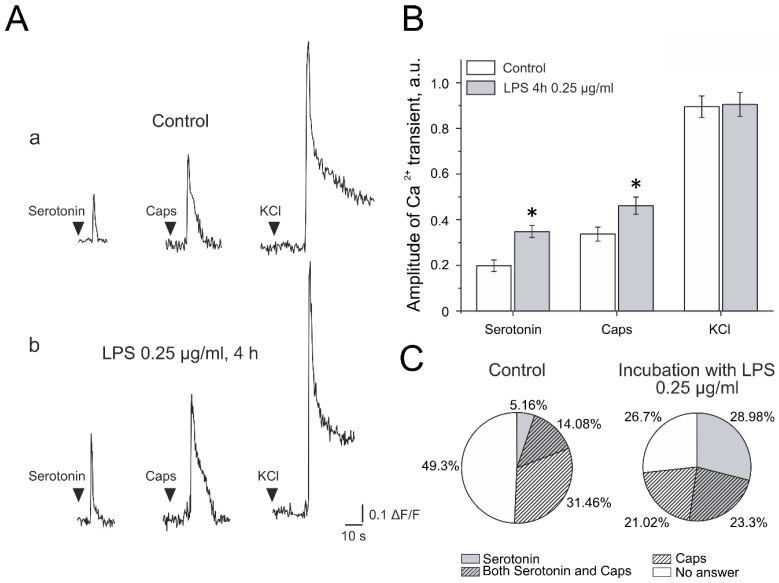
Effect of LPS on serotonin- and capsaicin-induced calcium signaling in rat trigeminal neurons. (**A**) Examples of recordings of fluorescence intensity (F − F0/F0) from Fluo-4 AM-stained TG neurons (**a**) in control conditions and (**b**) after 4 h of incubation in LPS-containing saline (0.25 μg/mL). (**B**) The mean amplitude of Ca^2+^ responses induced by applications (2 s) of 30 μM serotonin (Ser), 1 μM capsaicin (Caps), and 50 mM KCl in control conditions and after incubation with LPS. (**C**) The percentage of serotonin- and capsaicin-sensitive TG neurons in control conditions and after incubation with LPS. * *p* < 0.05 compared to initial value.

In our study, serotonin (30 μM) was used to activate 5-HT receptors, and capsaicin (1 μM) evoked responses mediated by TRPV-1 receptors (Figure 4A). Neurons were identified by their fluorescence response to KCl application (50 mM; 2 s). In control conditions, the application of serotonin (2 s) induced Ca^2+^ transients in 19% (41/213 cells, n = 4) of TG neurons, and capsaicin (2 s) induced Ca^2+^ transients in 46% (97/213 cells) of cells (Figure 4C). After 4 h incubation in F12 medium containing 0.25 μg/mL LPS, the percentage of capsaicin-responsive neurons did not change (44%, χ^2^ = 0.06, *p* = 0.806, 78/176 cells, n = 4). In contrast, the proportion of serotonin-responsive cells significantly increased to 52% (χ^2^ = 46.71, *p* = 0.0001, 92/176 cells). It was shown that 4 h of incubation in 0.25 μg/mL LPS significantly increased the mean amplitude of Ca^2+^ transients evoked by both agonists: serotonin (0.20 ± 0.03 a.u. in control vs. 0.35 ± 0.03 a.u. after incubation, *p* = 6.68) and capsaicin (0.34 ± 0.03 a.u. in control vs. 0.46 ± 0.04 a.u. after incubation, *p* = 0.002; Figure 4A,B). The mean amplitude of the response to KCl application did not change: 0.90 ± 0.05 in the control and 0.91 ± 0.05 after incubation with LPS (*p* = 0.91).

Furthermore, it was shown that, after incubation in LPS, the number of TG neurons sensitive to both agonists increased (30/213 vs. 41/176 cells, n = 4, χ^2^ = 5.48, *p* = 0.0192, Figure 4C); at the same time, the percentage of non-responsive cells decreased almost twice (105/213 vs. 47/176 cells, χ^2^ = 20.66, *p* = 0.00000548).

## 3. Discussion

According to the current understanding, the activation of the peripheral meningeal structures of the TVS is responsible for generating the nociceptive signal in migraine. The processing of this signal and the development of somatic symptoms (e.g., photophobia and skin allodynia) are caused by its transmission to the second-order neurons of the central trigeminal nucleus [1,3,7,37]. Unmyelinated C fibers, which are part of the meningeal afferents of the trigeminal nerve, can produce various pro-nociceptive agents (e.g., CGRP, PACAP, VIP, and substance P). These substances then bind to their respective receptors, primarily located within Aδ fibers. This leads to the initiation of secondary intracellular biochemical cascades, nerve fiber sensitization, and amplified action potential generation [1,37]. The nociceptive activation of nerve fibers and their release of CGRP can lead to vascular changes, activation of satellite glial cells, and the altered activity of meningeal immune cells such as mast cells and macrophages [12,14,37,38].

To date, no studies have been conducted on experimental animal models describing the changes in nociceptive signaling associated with migraine in the TVS under conditions of induced systemic inflammation, including immune system activation in the early stages of ontogenesis during the prenatal period. Therefore, modeling systemic inflammation could be a promising approach for studying both peripheral inflammation in the dura mater and, potentially, the effects of central neuroinflammation. This is due to the fact that pro-inflammatory mediators from the periphery can penetrate the brain and affect neurons, including nociceptive ones [39,40,41,42]. Investigating nociceptive processes in the context of systemic inflammation could reveal relationships between inflammation, neuroinflammatory reactions, and nociceptive signaling in migraine.

It is well-established that inflammatory processes during fetal development can lead to delayed neurological disorders accompanied by deficits in cognition and sensory processing [43,44]. Analysis of patient medical data in several studies has revealed a positive correlation between exposure to various stressful conditions in the prenatal period and the subsequent onset of migraine in adults and children [42,43,44]. Prenatal factors associated with the subsequent onset of migraine include exposure to bacterial infections, tobacco smoke, and ethanol. These factors cause the release of reactive oxygen species (ROS) and can trigger the development of hypersensitivity to neuroinflammation and oxidative stress in the fetal brain [45,46,47,48].

LPS administration to rodents during pregnancy leads to elevated levels of pro-inflammatory cytokines and oxidative stress markers in various areas of the offspring’s brain (including the cerebellum, hippocampus, and cortex), as well as the manifestation of an anxiety-like behavioral phenotype [49,50,51]. Furthermore, we have previously demonstrated that creating an inflammatory challenge during the prenatal period in the form of maternal hyperhomocysteinemia, achieved through dietary interventions, results in blood–brain barrier permeability disorders in offspring [52], as well as elevated plasma CGRP levels and increased excitability of meningeal afferents of the trigeminal nerve and pronounced neuroinflammatory reactions in the dura mater [53], alongside a decrease in the cortical spreading depression generation threshold in the sensomotor cortex and pronounced migraine-associated behavioral traits such as mechanical allodynia and photophobia [35].

The mechanism of such disorders in offspring under maternal immune activation (prenatal pro-inflammatory exposures) may involve the epigenetic regulation of gene transcription [44,54], disruption of the switch from excitatory to inhibitory gamma-aminobutyric acid (GABA) function in the early stages of postnatal ontogenesis (GABA shift) [55,56], abnormal expression of NMDA receptors, especially in the striatum and cortex [57], and the development of synaptic deficits (especially in the cortex) [58]. Within the TVS, maternal immune activation may lead to long-term sensitization and hyperactivity, possibly due to the enhanced expression of nociception-associated receptors (e.g., purinergic, TRPA1, and TRPV1) [59], changes in glial cells’ phenotype under the influence of pro-inflammatory mediators [60], and dysregulation of descending pain modulatory pathways (e.g., from the periaqueductal grey matter and the rostral ventromedial medulla), influencing second-order nociceptive neurons in the TNC [55,61].

The administration of LPS appears to amplify nociceptive behavioral responses. In a model of post-traumatic trigeminal neuropathy, for example, local administration of LPS has been shown to enhance orofacial nociceptive responses and increase IL-1 mRNA expression in the TG [29]. In a neuropathic pain model caused by sciatic nerve ligation, intrathecal administration of a selective toll-like receptor 4 (TLR-4) antagonist prevented the development of hyperalgesia and mechanical hypersensitivity [62]. In an orofacial postoperative pain model, injection of the TRR-4 antagonist into the TG prevented the development of facial heat and orofacial mechanical hyperalgesia [26]. Similarly, local administration of LPS to the joint area and paw pads has been shown to decrease the mechanical thresholds and cause hyperalgesia [30,31,32]. However, studies investigating the effect of LPS on nociceptive responses have mainly focused on models of inflammatory and post-traumatic pain. Conversely, no studies have assessed the impact of inflammation induced by LPS administration on nociceptive responses and the nociceptive process in migraine.

In our study, maternal immune activation via an LPS injection on day 15 of fetal development resulted in decreased mechanical sensitivity thresholds and more pronounced behavioral changes in response to chronic NTG administration (as part of a migraine model) in offspring aged 30–45 days. It also resulted in mast cell degranulation in the dura mater. When conducting the Light–Dark Transition test, aimed at identifying photophobic behavior, we found that, in the migraine model with acute LPS administration, the values of photophobic behavior did not differ from those of the control group. In contrast, in the migraine model with prenatally induced systemic inflammation caused by LPS administration, a significant reduction in the time spent in the light chamber before and after NTG injection was observed on day 9 of drug administration, compared to the control group, indicating more pronounced photophobia in the prenatal LPS group. Evaluation of the locomotor activity showed that in the experimental groups (control group, acute LPS, and prenatal LPS), the count of locomotor acts such as transitions between chambers and rearing tended to decrease by day 9 of NTG administration in a similar manner; however, the NTG injection itself did not lower the number of locomotor acts.

Administering LPS to rodents is a widely used and reliable approach for modeling systemic inflammation, local inflammatory syndromes, and neuroinflammatory processes [63,64,65]. In LPS-induced inflammation, LPS binds to TLR4, which is widely expressed in neurons, microglia, macrophages, astrocytes, and vascular endothelium, including the cerebral endothelium [64,66]. The binding of LPS to TLR4 leads to homodimerization, activating intracellular secondary mediator cascades and the subsequent transcription of genes encoding pro- and anti-inflammatory mediators (e.g., cytokines, chemokines, and prostaglandins) [64,66].

In response to LPS, a family of pro-inflammatory cytokines is synthesized and released (IL-1, IL-6, and TNF-α) [63,64,67]. The pro-inflammatory effect of LPS is assumed to be due to an intracellular increase in reactive oxygen species (ROS) through the activation of the NADPH oxidase enzyme. ROS then serve as secondary messengers, enhancing the expression of pro-inflammatory marker genes in microglia and other cell types [17,18,19]. TLR-4 expression has been detected in the TG and nociceptive afferents of the trigeminal nerve [68], indicating the potential for LPS-induced changes in nociceptive signaling associated with migraine.

To identify potential LPS-induced mechanisms contributing to the development of nociceptive behavior, we administered a single acute dose of LPS to adult rats. Then, we analyzed their nociceptive behavior and recorded the trigeminal nerve activity in rat hemiskull preparations. It was previously shown that a single postnatal administration of LPS leads to both immediate and long-term inflammatory reactions, including an increase in the TNF-α concentration in the brain 10 months after administration [69]; an increase in IL-18 in the hippocampus, frontal cortex, and cerebellum 10 months after administration; stable changes in exploratory behavior and cognitive functions [70,71]; and long-term activation of microglia [72]. Immediate inflammatory responses to LPS administration include increased concentrations of TNF-α, IL-1β, IL-6, IL-4, and NF-κB in various brain regions (hippocampus, substantia nigra, cortex, cerebellum, and striatum) several hours (3–6 h) after injection [70,73,74], as well as peripheral inflammatory responses [41,75,76]. Therefore, we performed an acute LPS injection and chose 3 h after LPS administration as the time point for further electrophysiological, histological, biochemical, and behavioral experiments.

CGRP is a key molecule in the formation of trigeminal nerve afferent sensitization and an increase in the nociceptive activity in migraine [1,9]. It has been shown that level of CGRP in plasma increases in patients with chronic migraine both during headache attacks and between attacks [77,78]. In the current study, we found that, in the control group of animals with chronic administration of NTG, the content of CGRP in blood plasma tended to increase. Meanwhile, in the acute LPS group, a significant increase in CGRP concentration was observed after chronic administration of NTG, compared to the baseline level.

Serotonin is a key player in peripheral nociceptive signaling during migraine. Its receptors in the meningeal endings of the trigeminal nerve contribute to peripheral sensitization and hyperalgesia development [11,79,80]. During mast cell degranulation, serotonin is released into the meningeal tissue, where it binds to ionotropic 5-HT3 serotonin receptors located in nerve endings, leading to the nociceptive activation of Aδ- and C-fibers [11,13,80]. Activation of these receptors also leads to the release of CGRP, which activates pro-nociceptive trigeminal afferent fibers and is thought to cause long-term headache in migraine [79,81].

Our data indicate that, in animals with systemic inflammation induced both by prenatal and by acute postnatal LPS injection, a more pronounced pro-nociceptive activation of trigeminal nerve afferents in response to serotonin application was observed, which potentially indicates the involvement of serotonergic mechanisms in the intensification of migraine with LPS-induced neuroinflammation.

The role of 5-HT3 receptors in the inflammatory process was demonstrated in a model of temporomandibular joint (TMJ) inflammation, where 5-HT3 receptor blockers reduced orofacial nociceptive behaviors evoked by formalin injection into the masseter muscle [82]. In a model of persistent TMJ inflammation evoked by the injection of complete Freund’s adjuvant, the expression of 5-HT2A and 5-HT3 receptors in TG increased during TMJ inflammation. It has been shown that these receptor mRNAs increase in primary sensory neurons after peripheral inflammation [83,84,85]. Inflammation in the TMJ can cause the increased excitability of TG neurons that receive incoming signals from the masticatory muscles, and it is assumed that increased peripheral expression of 5-HT2A and 5-HT3 during inflammation of the mandibular joint may play a role in increasing the excitability of primary sensory afferents [83].

In a human blood monocyte cell culture under conditions of preliminary LPS application, there was a serotonin-dependent increase in IL-1β, IL-6, and IL-8/CXCL8. Activation of other serotonin receptor subtypes (5-HT4 and 5-HT7) increased intracellular cyclic adenosine monophosphate levels and IL-1β, IL-6, and IL-12 secretion [86]. Meanwhile, creating an inflammation model by injecting bee venom into a rat’s paw surface led to the increased mRNA expression of 5-HT3, 5-HT4, 5-HT7, and 5-HT2 in dorsal root ganglion (DRG) neurons [85]. Interestingly, LPS can influence the activity of the serotonin transporter peripherally, as demonstrated in intestinal endothelial cells, and centrally, as demonstrated in the prefrontal cortex and adjacent nucleus. Thus, LPS can affect the rate of serotonin metabolism [87,88].

It was shown that LPS can directly affect neuronal and non-neuronal cells, causing the release of cytokines, mainly TNF-α, which has been demonstrated in numerous studies on DRG neurons, both in vitro and in vivo [89,90,91].

Studies on TG tissue have shown that local LPS administration leads to increased TNF-α and IL-1β levels and TRPV1 expression [29,92,93]. Studies using TG cell cultures indicate that LPS treatment leads to neuronal sensitization and the mobilization of intracellular and extracellular calcium in a TRPA1-dependent manner [94]. It also leads to increased CGRP release in response to capsaicin application, which indicates the LPS-induced sensitization of TRPV1 receptors in TG neurons [95,96]. Such sensitization of TG neurons is thought to involve the binding of LPS to its target receptor, TLR4. This leads to increased NFκB phosphorylation, affecting the activity of secondary intracellular mediators and modulating the activity of receptors [94,95,96,97]. Interestingly, in TG neuron cultures, the increased CGRP release induced by LPS was prevented by pretreatment with TRPA1 or TRPV1 antagonists [97]. However, no studies have analyzed the role of the serotonergic system in sensory ganglia in response to inflammation.

In our experiments on neurons isolated from the TG, LPS pretreatment increased calcium transients in response to serotonin and capsaicin application. We observed an increase in the number of neurons responding to serotonin by more than two-fold and in the number of neurons responding to both serotonin and capsaicin by 1.5-fold, which may indicate the upregulation of 5-HT_3_ receptors in TG during pro-inflammatory conditions [83,84,85]. The amplification of TRPV1-mediated calcium signals may be associated with the upregulation of TRPV1 receptors [28,92,93] and serotonin effects [98]. Additionally, 5-HT-induced sensitization of TRPV1 channels can lead to increased CGRP release from TG neurons [81], which may contribute to inflammatory pain upon LPS administration.

Furthermore, our study revealed an increased degranulation of mast cells in the meninges under conditions of prenatally induced inflammation and after acute LPS administration. This verifies both methods of LPS administration as effective approaches for investigating neuroinflammatory processes in the dura mater. Mast cells closely interact with the peripheral afferents of the trigeminal nerve to form a “neuroimmune synapse,” wherein the activation of trigeminal afferent fibers leads to the release of CGRP and the degranulation of mast cells, which in turn release pro-inflammatory cytokines and serotonin. These substances contribute to the additional activation of nerve fibers [12,13,39,99]. Neuroinflammatory reactions in the dura mater significantly contribute to peripheral sensitization by creating a “vicious circle” of nociceptive signal amplification via interaction between immune cells and nerve endings [11,12,14,15].

The mechanism of the LPS-dependent degranulation of meningeal mast cells may involve LPS-induced ATP release (as demonstrated in astrocytes) [100], which can lead to mast cell destabilization through P2X7 receptor activation, as previously demonstrated [11,12]. Our results are consistent with previous studies showing that LPS can affect non-neuronal cells and potentially alter the phenotype of nociceptive neurons, influencing the manifestations and intensity of pain syndromes [100,101].

Thus, our study for the first time evaluated LPS-dependent nociceptive mechanisms in migraine. We established that LPS enhances mechanical allodynia and photophobia in a chronic NTG-induced migraine model. We observed the LPS-induced enhancement of serotonergic pro-nociceptive effects in trigeminal nerve afferents and TG neurons, as well as the degranulation of meningeal mast cells, using different methods of LPS administration. These findings can contribute to our fundamental understanding of the molecular and cellular mechanisms of nociception associated with migraine and inflammatory orofacial pain syndromes.

This study has several limitations. First, animals of different ages were used for behavioral and electrophysiological experiments compared to Ca^2+^ imaging in cultured TG neurons. This discrepancy, necessitated by the technical requirements of each method, may introduce biological heterogeneity. Second, while our functional data suggest the involvement of specific receptors, molecular confirmation of 5-HT_3_ and TRPV1 receptor expression in the TG is required. This analysis is planned for future investigations.

## 4. Materials and Methods

### 4.1. Animals

The experiments were conducted on Wistar rats, aged 30–65 days (P30–65), obtained from the vivarium of the Department of Human and Animal Physiology, Kazan Federal University, Kazan, Russia. The animals were kept in a room with a 12 h light/12 h dark cycle, with the lights turned on every day at 6:00 a.m.; the room temperature was maintained at ~22 °C, and the air humidity was controlled. The animals had ad libitum access to water and food.

**Figure 5:**
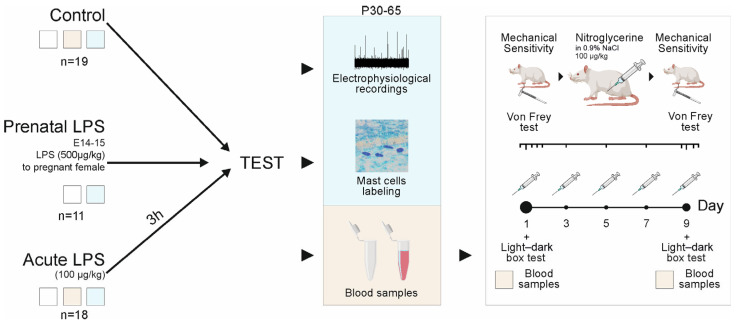
Experimental protocol diagram. Three groups of animals were used in the study (control, prenatal LPS, acute LPS). The control group did not receive LPS, (n = 19); the prenatal LPS group contained a model of prenatal inflammation consisting of the male offspring from females exposed to LPS on days 14–15 of pregnancy (i.p., 500 μg/kg, n = 11); the acute LPS group contained a model of acute inflammation created by a single injection of LPS (i.p., 100 μg/kg, n = 18) 3 h before the experimental protocols. Some of the animals in each group were used for electrophysiological and histological studies (blue square). The other part, after blood sampling for biochemical studies (pink square), was used in a series of behavioral tests during episodic and chronic nitroglycerin administration. The color of the square indicates the tests that were performed in the presented group of animals. The age of the animals in the groups was P30–65, and the animals were male.

The study used the following experimental animal groups all consisting of Wistar rats: (1) sham group that did not receive any drugs; instead of nitroglycerin (NTG), they were injected with 0.9% NaCl (saline) (n = 4); (2) control group that did not receive LPS, (n = 19); (3) acute LPS group with a model of acute inflammation created by a single injection of LPS (i.p., 100 μg/kg, n = 18); (4) prenatal LPS group, including the offspring of females, who were exposed to LPS on days 14–15 of pregnancy (i.p., 500 μg/kg, n = 11) (Figure 5).

Male rats were used in the behavioral and electrophysiological experiments to avoid any instability induced by hormonal changes during the estrous cycle in females.

All experimental protocols were performed in accordance with the European Community Council Directive of 22 September 2010 (2010/63/EEC) and approved by the Local Ethical Committee of Kazan Federal University (protocol No. 56 of 19 September 2025). All efforts were made to minimize the number of animals used in the experiments.

### 4.2. A Model of Systemic Inflammation Induced by Injection of LPS

Maternal immune activation (MIA) appears to act as a “disease primer” [33] to make an individual more susceptible to the effects of genetic mutations and environmental exposures in triggering disease-related symptoms later in life [34]. In this study, MIA was utilized to create a greater susceptibility to migraine and promote migraine-related neuroinflammation and nociceptive behavior. Pregnancy was achieved by placing male rats with female rats at a ratio of 1:2 for a period of 7 days. Fertilization was confirmed by microscopy of a stained vaginal smear. To cause systemic inflammation leading to neuroinflammation, we used a validated LPS model on rodents [63,64,65]. LPS (trade name Pyrogenal, N. F. Gamaleya Research Institute of Epidemiology and Microbiology, Moscow, Russia) at a concentration of 500 μg/kg was administered intraperitoneally to pregnant rats [102] on the 15th day of pregnancy (GD 15), approximately corresponding to the second trimester of human pregnancy [34]. The dosage of LPS administered was selected based on an analysis of the literature to avoid spontaneous fetal loss and stillbirth [33,34,102,103,104]. Further studies were conducted on the male offspring at the age of P30–65 (prenatal LPS group).

In the acute LPS group, inflammation was induced by a single intraperitoneal injection of LPS (100 μg/kg) to rats in the postnatal period (P30–65). The dosage was selected based on an analysis of the literature to avoid pronounced toxic effects and severe symptoms of parkinsonism [105,106,107]. Three hours after LPS administration, behavioral, histological, or electrophysiological studies were performed.

### 4.3. Model of Episodic and Chronic Migraine

NTG (Ozon Pharmaceuticals, Zhigulevsk, Russia) was used to create a model of acute or chronic migraine in rats, testing the mechanical sensitivity and photophobia [32,105]. NTG (10 mg/kg in 0.9% NaCl solution) was repeatedly administered intraperitoneally (i.p.), five times every second day for nine days (days 1, 3, 5, 7, and 9). Mechanical hypersensitivity was measured 2 h before (pre-injection/basal response) and 2 h after (post-injection response) NTG injections [108,109]. The development of photophobia was assessed using the dark–light box test before and after the first and last injections of NTG. In the control group, 0.9% NaCl solution was introduced as a vehicle. All behavioral experiments were conducted at the same time and started at approximately 10 a.m.

### 4.4. Analysis of Mechanical Sensitivity Using Von Frey Test

Mechanical sensitivity was assessed with a series of calibrated von Frey filaments (Ugo Basile, Varese, Italy) with a range of 0.008 to 8 g of the target force, which corresponds to 2.53–61.7 g/mm^2^ pressure. First, 30 min before the test, the rat was placed in an individual transparent box with a mesh floor. The mechanical withdrawal thresholds were determined according to the up-and-down method [35,36,110]. Von Frey filaments were applied by approaching the plantar surface of the paw from the underside of the mesh stand. We always started by testing with a 0.4 g (3.61 g/mm^2^) filament. In all cases, the tip of the filament was pressed against the plantar surface of one hind paw and maintained for 1–3 s. A response was defined as withdrawal, shaking, or licking of the paw. In the absence of a response, a heavier filament (up) was tried after 10 s, and in the presence of a response, a lighter filament (down) was tested. This pattern was followed for a maximum of four filaments following the first response.

The mechanical sensitivity was measured 1 h before (pre-injection/baseline response) and 2 h after (post-injection response) NTG injections, except on the first day. On the first day of NTG administration, mechanical thresholds were measured 1, 2, and 3 h after injection for a detailed analysis of mechanical sensitivity in the episodic migraine model [108], while on days 3, 5, 7, and 9 of NTG administration, testing was performed only once, 3 h after injection. In addition, a separate group of animals (n = 4) received saline injections in the same manner to form a sham group, and the mechanical thresholds of the hind paws were also measured as described above.

### 4.5. Study of Photophobic Behavior

The Light–Dark Transition test was used to measure photophobia [36]. The light–dark box consists of two equally sized chambers: one illuminated (150 lux) and one darkened (1–2 lux) with a size of 40 cm × 20 cm × 40 cm/each connected with a passageway 7 cm × 7 cm (OpenScience, Moscow, Russia) equipped with a video system Sony SSC-G118 (Tokyo, Japan). The rats were placed in the light compartment and allowed to explore the apparatus for 3 min. We measured the time spent in the light chamber and the locomotor activity of animals (transitions between chambers and rearing).

Testing was performed prior to NTG administration (baseline, pre-injection value) and 2 h after injection (post-injection value) on days 1 and 9 of migraine modeling (after the first and fifth NTG injections).

### 4.6. Electrophysiological Recording of Action Potentials of Trigeminal Nerve Afferents

The activity of trigeminal afferents was recorded using isolated rat hemiskulls with intact dural innervation obtained from rats P40–45, as described previously [11,36,111]. Prior to decapitation, the animals were deeply anaesthetized using isoflurane (Abbott Laboratories, Chicago, IL, USA). The rat skull after decapitation was carefully cleaned of all cranial muscles; then, it was divided along the sagittal section into two hemiskulls, and the brain was removed from the skull. The distal part of the nervus spinosis that innervates the receptive field around the middle meningeal artery was isolated from the hemiskull and placed inside the recording electrode. The preparations were perfused with an artificial cerebrospinal fluid containing 120 mM NaCl, 2.5 mM KCl, 2 mM CaCl_2_, 1 mM MgCl_2_, 11 mM glucose, 1 mM NaHPO_4_, and 24 mM NaHCO_3_ under constant oxygenation at 95% O_2_/CO_2_, a pH range of 7.2–7.35, and at 20–25 °C. The action potentials (APs) of the trigeminal nerve were recorded using a DAM 80 amplifier and digitized on a PC with an NI PCI6221 board (National Instruments, Austin, TX, USA). The signals were visualized and analyzed using WinEDR v.3.2.7 software (University of Strathclyde, Glasgow, UK). The AP frequency was measured and analyzed using the DoClust application in the MATLABver. 9.8 (R2020a) package (MathWorks, Natick, MA, USA) and OriginPro 2015 (OriginLab Corporation, Northampton, MA, USA) software. We used native (control) animals as well as animals treated with acute LPS injection (100 μg/kg) in the electrophysiological experiments. First, the baseline control recordings were taken for 20 min previous to drug application. Then, serotonin 20 μM (Sigma-Aldrich, St. Louis, MO, USA) and capsaicin 1 μM (Sigma-Aldrich, St. Louis, MO, USA) were applied in separate experimental series to study the activity of meningeal trigeminal afferents. Substances were applied through gravitation-driven perfusion system at the receptive field of trigeminal afferents.

### 4.7. Toluidine Blue Staining of Meningeal Mast Cells

Mast cell degranulation was assessed using the histological method of staining for the hemiskull with Toluidine Blue [11,36,112]. Intact skulls were placed in paraformaldehyde (4% solution) for 12 h. Before isolating the meninges, the skulls were washed in a phosphate-buffered saline solution of the following composition (mM): 137 NaCl, 2.7 KCl, 10 Na_2_HPO_4_, 1.8 K_2_HPO_4_. The isolated meninges were fixed on a glass slide. Staining with Toluidine Blue lasted 10 min, and then the fixed preparations were washed with distilled water and dehydrated with ethyl alcohol (95–99%). Images were acquired with 20× magnification. The degree of degranulation was assessed visually; the calculation was carried out as the percentage of the total number of cells (at least 100).

### 4.8. Ca^2+^ Imaging of Trigeminal Ganglion Neuron Cultures

Primary culture of TG cells was prepared using TG of rats (P7-P12), as described previously [113]. The ganglia were removed after rat decapitation and placed in cold F12 medium. Cells were dissociated using an enzymatic cocktail containing 0.25 mg/mL trypsin, 1 mg/mL collagenase, and 0.2 mg/mL DNAse. The dissociation of TG was made in shaker at 37 °C for 25 min. Dissociated cells were placed on coverslips coated with poly-L-lysine and incubated for 24 h before the experiments.

To perform Ca^2+^ imaging, Fluo-4 AM (1 μM, Invitrogen, Carlsbad, CA, USA) was used, as described previously [53].

To detect and measure changes in intracellular calcium levels, cultured TG cells were stained using Fluo-4 AM dye (Invitrogen, Carlsbad, CA, USA) in the presence of pluoronic acid (0.02%) at 37 °C for 30 min in the absence of light. Before the experiments, the cells were washed out with an extracellular solution for 5–10 min.

Fluorescence visualization of stained cells was performed using an Axio Observer.D1 microscope (Carl Zeiss, Jena, Germany), an excitation filter (BP 450–490 nm), a beam splitter (FT 510 nm), and an emission filter (LP 555 nm). Fluorescence images were recorded using an AxioCam MRm high-speed camera (Carl Zeiss, Germany).

All substances were applied using a gravity-controlled perfusion system (ALA Scientific Instruments, Westbury, NY, USA). At the end of each experiment, KCl (50 mM, 2 s) was applied to initiate membrane depolarization and differentiate between neuronal and glial cells. Fluorescence images were converted into signals using ImageJ 1.52s software (NIH, Bethesda, MD, USA), and the fluorescence intensity was estimated in arbitrary units (a.u.). The peak fluorescence (amplitude) was expressed as the relative change in fluorescence (F − F0)/F0 for each cell of each frame, where F is the peak fluorescence of a cell, and F0 is the background fluorescence close to a given cell. Any changes in fluorescence intensity that were beyond the flat baseline at the time of agonist application and had a characteristic asymmetric shape with a rapid rise and slow decline were considered as Ca^2+^ responses. The fluorescent response to KCl was used to normalize the amplitudes of the responses of TG neurons for all substances used in the current study.

### 4.9. Plasma CGRP Concentration

Blood sampling in rats was performed using gum incision. The samples were then mixed with an anticoagulant in tubes and centrifuged at 1500 rpm for 15 min. The supernatant was separated and stored at −40 °C until ELISA analysis. In the control group, blood collection was performed before and after chronic NTG administration—at day 9 after the last injection. In the acute LPS group, the CGRP levels were measured before and 3 h after LPS administration and after chronic NTG injections.

The concentrations of CGRP were determined using a rat-specific ELISA kit (CEA876Ra ELISA Kit for Calcitonin Gene Related Peptide, CGRP, Cloud-Clone Corp., Katy, TX, USA), according to the manufacturer’s instructions, and the samples were added to the corresponding micro-ELISA strip-plate wells. Specific horseradish peroxidase (HRP)-conjugated antibodies were added to each sample for incubation, followed by chromogen solutions A and B for coloring, and finally, a stop solution was added to terminate the reaction, changing the visible color from blue to yellow. The amount of bound HRP conjugates and the intensity of the color are inversely proportional to the concentration of the CGRP in the sample. The optical density was calculated using a microplate spectrophotometer (Multiskan FS, Thermo Fisher Scientific, Waltham, MA, USA) at a wavelength of 450 nm. Each sample was analyzed through a calibration curve, and the mean concentrations were calculated and expressed as pg/mL.

### 4.10. Statistics

Data analysis was performed using the MATLAB package (MathWorks, Natick, MA, USA) and Origin Pro 2015 (OriginLab Corporation, Northampton, MA, USA). The Shapiro–Wilk test was used to determine the normality of the dataset. The nonparametric Mann–Whitney U-test was used to compare the two independent groups. The two-sided Wilcoxon rank sum test for paired samples or the paired *t*-test were used to assess the effect of the compounds in the same preparation. The chi-square test was used for Ca^2+^ imaging data to compare the percentage of the responding cells in the different groups. Differences were considered statistically significant at *p* < 0.05. All sample sizes were greater or equal to the suggested sample size calculated in Origin Pro (power and sample test for paired or two-sample data) for 0.8 desired power to avoid Type II error [114].

## Data Availability

The original contributions presented in this study are included in the article. Further inquiries can be directed to the corresponding authors.

## Abbreviations

The following abbreviations are used in this manuscript:

TVS	trigeminal vascular system
LPS	Lipopolysaccharide
NTG	Nitroglycerin
CGRP	calcitonin gene-related peptide
TRPV1	transient receptor potential vanilloid 1
ATP	adenosine triphosphate
TNC	trigeminal nucleus caudalis
AP	action potential
5-HT	5-hydroxytryptamine
PACAP	pituitary adenylate cyclase-activating peptide
VIP	vasoactive intestinal peptide
ROS	reactive oxygen species
GABA	gamma-aminobutyric acid
TLR-4	toll-like receptor 4
TMJ	temporomandibular joint
TG	trigeminal ganglion
DRG	dorsal root ganglion
MIA	maternal immune activation
HRP	horseradish peroxidase
ELISA	enzyme-linked immunosorbent assay
